# Supplementary material for: From sequence to enzyme mechanism using multi-label machine learning
Source: BMC Bioinformatics. 2014 May 19;15:150. doi: 10.1186/1471-2105-15-150 (PMC4229970; doi:10.1186/1471-2105-15-150)
Supplement: Additional file 2 — Java code of ml2db. Additional file ml2db_code.tar.gz contains the Java source code to run the multi-label machine learning experiments and save the results to database. The code’s Javadoc is included. [file 1471-2105-15-150-S2.zip › additional file 2/ml2db/ml2db/doc/main/ml2db/mulan/learn/traintest/TrainTestOutputManager.html]

TrainTestOutputManager


JavaScript is disabled on your browser.


- Overview
- Package
- Class
- Use
- Tree
- Deprecated
- Index
- Help

- Prev Class
- Next Class

- Frames
- No Frames

- All Classes

- Summary:
- Nested |
- Field |
- Constr |
- Method

- Detail:
- Field |
- Constr |
- Method


main.ml2db.mulan.learn.traintest

## Class TrainTestOutputManager

- java.lang.Object
- - uk.ac.standrews.utils.main.Initialised
  - - main.ml2db.mulan.learn.crosseval.CrossOutputManager
    - - main.ml2db.mulan.learn.traintest.TrainTestOutputManager

- All Implemented Interfaces:
  :   uk.ac.standrews.utils.main.Initialisable

  Direct Known Subclasses:
  :   AlOutputManager

  ---

    

  ```
  public class TrainTestOutputManager
  extends CrossOutputManager
  ```

  Class

  Version:
  :   13 Jul 2011

  Author:
  :   Luna De Ferrari ldf(at)st-andrews.ac.uk

- - ### Field Summary

    - ### Fields inherited from class uk.ac.standrews.utils.main.Initialised

      `m_initialisationIsCorrect`
  - ### Constructor Summary

    Constructors

    Constructor and Description

    `TrainTestOutputManager(uk.ac.standrews.utils.main.database.DbManager resultsDbManager,
    java.lang.String resultsFolder,
    uk.ac.standrews.utils.main.database.Table table,
    TrainTestExperimenter experiment)`
  - ### Method Summary

    Methods

    | Modifier and Type | Method and Description |
    | --- | --- |
    | `void` | `closeConnection()` |
    | `TrainTestExperimenter` | `getExperimenter()` |
    | `void` | `logEvaluation()` |

    - ### Methods inherited from class main.ml2db.mulan.learn.crosseval.CrossOutputManager

      `getEvaluationMeasures, getEvaluationResultsTable, getExperimentDbManager, getResultsDirectory, getTable`
    - ### Methods inherited from class uk.ac.standrews.utils.main.Initialised

      `initialisationIsCorrect, setInitialisation, setInitialisationIsCorrect, setInitialisationNotCorrect`
    - ### Methods inherited from class java.lang.Object

      `equals, getClass, hashCode, notify, notifyAll, toString, wait, wait, wait`

- - ### Constructor Detail


    - #### TrainTestOutputManager

      ```
      public TrainTestOutputManager(uk.ac.standrews.utils.main.database.DbManager resultsDbManager,
                            java.lang.String resultsFolder,
                            uk.ac.standrews.utils.main.database.Table table,
                            TrainTestExperimenter experiment)
      ```
  - ### Method Detail


    - #### closeConnection

      ```
      public void closeConnection()
      ```


    - #### getExperimenter

      ```
      public TrainTestExperimenter getExperimenter()
      ```

      **Overrides:**
      :   `getExperimenter` in class `CrossOutputManager`


    - #### logEvaluation

      ```
      public void logEvaluation()
      ```

      **Overrides:**
      :   `logEvaluation` in class `CrossOutputManager`


- Overview
- Package
- Class
- Use
- Tree
- Deprecated
- Index
- Help

- Prev Class
- Next Class

- Frames
- No Frames

- All Classes

- Summary:
- Nested |
- Field |
- Constr |
- Method

- Detail:
- Field |
- Constr |
- Method
